# Biochemical and proteomic analyses of venom from a new pit viper, *Protobothrops kelomohy*

**DOI:** 10.1590/1678-9199-JVATITD-2021-0080

**Published:** 2022-04-11

**Authors:** Lawan Chanhome, Orawan Khow, Onrapak Reamtong, Taksa Vasaruchapong, Panithi Laoungbua, Tanapong Tawan, Sunutcha Suntrarachun, Siravit Sitprija, Supeecha Kumkate, Narongsak Chaiyabutr

**Affiliations:** 1 Snake Farm, Queen Saovabha Memorial Institute, The Thai Red Cross Society, Bangkok 10330, Thailand.; 2 Department of Research and Development, Queen Saovabha Memorial Institute, The Thai Red Cross Society, Bangkok 10330, Thailand.; 3 Department of Molecular Tropical Medicine and Genetics, Faculty of Tropical Medicine, Mahidol University, Bangkok 10400, Thailand.; 4 Department of Biology, Faculty of Science, Mahidol University, Bangkok 10400, Thailand

**Keywords:** Viperidae, Pit viper, Protobothrops, Venom proteomics, Snakebite, Antivenom, Cross-neutralization

## Abstract

**Background:**

A new pit viper, *Protobothrops kelomohy*, has been recently discovered in northern and northwestern Thailand. Envenoming by the other *Protobothrops* species across several Asian countries has been a serious health problem since their venom is highly hematotoxic. However, the management of *P. kelomohy* bites is required as no specific antivenom is available. This study aimed to investigate the biochemical properties and proteomes of *P. kelomohy* venom (PKV), including the cross-neutralization to its lethality with antivenoms available in Thailand.

**Methods:**

PKV was evaluated for its neutralizing capacity (ER_50_), lethality (LD_50_), procoagulant and hemorrhagic effects with three monovalent antivenoms (TAAV, DSAV, and CRAV) and one polyvalent (HPAV) hematotoxic antivenom. The enzymatic activities were examined in comparison with venoms of *Trimeresurus albolabris* (TAV), *Daboia siamensis* (DSV), *Calloselasma rhodostoma* (CRV). Molecular mass was separated on SDS-PAGE, then the specific proteins were determined by western blotting. The venom protein classification was analyzed using mass spectrometry-based proteomics.

**Results:**

Intravenous LD_50_ of PKV was 0.67 µg/g. ER_50_ of HPAV, DSAV and TAAV neutralize PKV at 1.02, 0.36 and 0.12 mg/mL, respectively. PKV exhibited procoagulant effect with a minimal coagulation dose of 12.5 ± 0.016 µg/mL and hemorrhagic effect with a minimal hemorrhagic dose of 1.20 ± 0.71 µg/mouse. HPAV was significantly effective in neutralizing procoagulant and hemorrhagic effects of PKV than those of TAAV, DSAV and CRAV. All enzymatic activities among four venoms exhibited significant differences. PKV proteome revealed eleven classes of putative snake venom proteins, predominantly metalloproteinase (40.85%), serine protease (29.93%), and phospholipase A_2_ (15.49%).

**Conclusions:**

Enzymatic activities of PKV are similarly related to other viperid venoms in this study by quantitatively hematotoxic properties. Three major venom toxins were responsible for coagulopathy in PKV envenomation. The antivenom HPAV was considered effective in neutralizing the lethality, procoagulant and hemorrhagic effects of PKV.

## Background

The Old World pit viper genus *Protobothrops* is recognized as widely distributed among other Asian genera including *Cryptelytrops*, *Garthius*, *Himalayophis*, *Ovophis, Parias*, *Peltopelor*, *Popeia*, *Trimeresurus*, and *Viridovipera* [1-3]. The newly described *Protobothrops kelomohy* from Chiang Mai and Tak Provinces, Thailand [4], is one of 15 species currently validated in the genus *Protobothrops*, which are *P. cornutus, P. dabieshanensis, P. elegans, P. flavoviridis, P. himalayanus, P. jerdonii, P. kaulbacki, P. manshenensis, P. maolanensis, P. mucosquamatus, P. sieversorum, P. tokaensis, P. trungkhanensis* and *P. xiangchengensis* [5, 6]. Within these validated species, a new distribution of *P. mucrosquamatus* has been recently reported in Nan Province, northern Thailand [7]. *Protobothrops* bites have constituted a serious health problem given that its highly hematotoxic venom causes a profoundly delayed coagulopathy, hypofibrinogenemia [8, 9], potent hemorrhagic shock effects, acute kidney injury, rhabdomyolysis and fatality [10-12]. *P. mucrosquamatus* has been widely recognized for its high medical importance (Category 1) in China and Taiwan, and moderate medical importance (Category 2) in India, Vietnam, Myanmar (Kachin State) and Laos [13]. In Taiwan, *P. mucrosquamatus* envenomation induced severe local effects presenting with swelling, hemorrhagic bulla, wound ecchymosis, cellulitis, tissue necrosis and compartment syndrome, of which the latter two were the main reason for surgical therapy [14]. Meanwhile, the observed systemic manifestations included leukocytosis, thrombocytopenia, coagulopathy, rhabdomyolysis and acute renal injuries [10]. Transient acute renal impairment progressing to life-threatening airway obstruction was also reported in *P. mucrosquamatus* envenoming [15]. These clinical manifestations after envenomations may pose a serious or potentially deadly emergency, and early therapeutic administration of antivenom is necessary if severe envenomation is suspected. 

In Thailand, monovalent or polyvalent hematotoxic antivenom is commonly administered during envenomation caused by the snakes of families Viperidae. Antivenom is produced from immunized (Fab´2) horses with one of the following viperid snake venoms: *Trimeresurus albolabris* venom (TAV) for *Trimeresurus albolabris* monovalent antivenom (TAAV); *Daboia siamensis* venom (DSV) for *Daboia siamensis* monovalent antivenom (DSAV); *Calloselasma rhodostoma* venom (CRV) for *Calloselasma rhodostoma* monovalent antivenom (CRAV) and TAV, DSV, and CRV for hemato polyvalent antivenom (HPAV). In spite of the widespread distribution of *Protobothrops sp*. in Asia, the venom of *P. kelomohy* (PKV) is not one of the species utilized for antivenom production. Adequate snakebite treatment is dependent on the ability of the antivenoms immunologically binding with venom components to reverse the pathological venom-induced symptoms. In addition, antivenom is known as the only definitive treatment in snakebite envenomation, but the availability of specific, effective antivenom remains limited in many areas [16], especially antivenom for *Protobothrops* species. Therefore, knowledge on venom composition and inter- and intra-specific venom variability should be investigated for assessment of a comparative analysis for the cross-neutralized immunoreactivity profile of antivenom efficacy from available viperid antivenom against PKV. 

Very few data are available on the biochemical properties and characterization of PKV. Therefore, this study aimed to elucidate the biochemical properties of PKV to support the clinical observation of its envenoming effects. Given the need to study the variation of PKV composition, including venom protein antigenicity and immunological reactivity with other specific antivenoms from other viperid snakes, the present study investigated the protein composition of the PKV and compared the para-specific antivenom through cross-neutralization antivenom. The knowledge of the cross-neutralization by the conventional antivenoms available in Thailand will provide valuable information for the subsequent comparison of antivenoms available in other countries, results that may be of wider interest. 

## Materials and methods

### Snake collection

Two *Protobothrops kelomohy* snakes were captured from Chiang Mai, northern Thailand and transferred to the Snake Farm of Queen Saovabha Memorial Institute (QSMI) in Bangkok. Snakes were housed individually in secure locked plastic cages, equipped with a hiding box and a water bowl, where they were fed mice once weekly [17]. 

### Snake venom preparation

Venom was collected through the bite of the snake on a parafilm membrane covering a glass vessel. The fresh venom was then pooled, immediately frozen at -20^0^C and lyophilized by Freeze Dryer Model FDL-10N-50-TD (MRC Scientific Instruments). The lyophilized venom was stored at -20 ^0^C until use. The venoms of *Trimeresurus albolabris* (TAV), *Daboia siamensis* (DSV) and *Calloselasma rhodostoma* (CRV) used for experimental comparison were supplied by the Snake Farm at QSMI.

### Antivenoms

Four antivenoms manufactured by QSMI were used: *Trimeresurus albolabris* monovalent antivenom (TAAV; Batch no. TA00119); *Daboia siamensis* monovalent antivenom (DSAV; Batch no. WR00117); *Calloselasma rhodostoma* monovalent antivenom (CRAV; Batch no. CR 00218) and hemato polyvalent antivenom (HPAV; Batch no. HP00118). All antivenoms were used within their shelf-lives. The freeze-dried Fab´2 antivenom was obtained from horses immunized with corresponding venoms, namely TAV, DSV, and CRV for TAAV, DSAV, and CRAV, HPAV against those of TAV, DSV and CRV. For the studies of neutralization capacity, each vial of antivenom was reconstituted with 10 mL of sterile water. According to the manufacturer’s guideline for leaflet, one milliliter of reconstituted antivenom can neutralize approximately 1.0 mg of TAV for TAAV; 1.0 mg of DSV for DSAV; 2.2 mg of CRV for CRAV; 1.0 mg of TAV, 0.73 mg of DSV, and 2.1 mg of CRV for HPAV. 

### Determination of lethal toxicity (LD_50_)

The outbred male ICR mice weighing 18-20 grams were supplied by the National Laboratory Center, Mahidol University. The lethal toxicity was determined through intravenous injection of 0.2 mL of serial 1.2-fold dilution of venom solution into the caudal vein of mice. The control group received a normal saline solution. Five mice were tested at each venom dose. The survival/death ratio was recorded after 24 h. The median lethal dose (LD_50_) was calculated by Reed and Muench [18]. 

### Determination of neutralizing capacity of antivenom

The median effective dose (ED_50_) was determined by pre-incubation of venom and antivenom [19]. Firstly, the mixtures containing 4 LD_50_ of PKV solution and the varying amounts of each antivenom (TAAV, DSAV, CRAV, and HPAV) were incubated at room temperature for 60 min, then centrifuged at 10,000 rpm for 5 min. Subsequently, 200 μL of the supernatants was intravenously injected into the outbred male ICR mice weighing 18-20 grams (n = 5 per dose of venom-antivenom mixture). All antivenom was unable to protect any mouse challenged with the 4 LD_50_ venom dose. Therefore, the dosage of PKV was adjusted to 3 LD_50_ tested with HPAV and DSAV, 2.5 LD _50_ tested with TAAV and 2 LD_50_ tested with CRAV in this experiment. The survival/death ratio was recorded after 24 h. The ED_50_ was defined as the dose of antivenom (µL) protecting 50% of mice against 2-3 LD_50_ of venom, calculated by the method of Reed and Muench [18]. The neutralization capacity of antivenom (ER_50_) was expressed as the amount of venom (mg) neutralized by 1 mL of antivenom at a 50% survival rate (mg/mL). The animal experimental protocol was approved by the Ethics Committee for Animal Care and Use at the Queen Saovabha Memorial Institute (approval number QSMI-ACUC-02-2018) in accordance with the guideline of the National Research Council of Thailand. 

### Determination of enzymatic activities

### Protease activity

Protease or proteolytic activity (PRO) was determined using casein as the substrate [20]. The mixture of 0.5 mL of 2% casein and 50 µL of venom solution was incubated at 37°C for 1 h. The reaction was terminated by adding 0.5 mL of 5% trichloroacetic acid, then centrifuged at 10,000 rpm for 5 min. The supernatant (0.4 mL) was allowed to react with 1 mL of 0.5 M sodium carbonate and 0.2 mL of Folin & Ciocalteau’s phenol reagent (1:5, v/v) at 37°C for 30 min. The developed color was measured at the absorbance of 660 nm. The activity of the protease is compared to a standard tyrosine curve in micromoles (µM). One unit of proteolytic activity was defined as the number of tyrosine equivalents released from casein per minute. The specific activity was expressed as units/mg/min.

### *Phospholipase A*_*2*_ *activity*

Phospholipase A_2_ activity (PLA_2_) was measured using the modified method [21]. Fifty microliters of venom solution was mixed with the substrate containing 50 µL of 3 mM 4-nitro-3-(octanoyloxy) benzoic acid and 0.5 mL of 10 mM CaCl_2_ in 10 mM Tris buffer (pH 8.0). The mixture was incubated at 37°C for 20 min, then the reaction was terminated with 50 µL of 2.5% Triton X-100. The absorbance was recorded at 425 nm. The PLA_2_ activity is defined as the amount of enzyme that caused a substrate absorbance change of 0.1 AU equivalent to 25.8 nmoles of chromophore release. 

### L-amino acid oxidase activity

The L-amino acid oxidase activity (LAAO) was determined with the modified technique [22]. Twenty-five microliters of 0.007% horseradish peroxidase and 50 μL of venom solution were added to the substrates containing 0.5 mL of 200 mM Triethanolamine buffer (pH 7.6), 0.1% L-leucine and 0.0065% o-dianisidine. The mixture was read at 30-second intervals for 3 min at 426-nm absorbance. One unit of LAAO activity was arbitrarily defined as the amount of venom that caused an increase of 0.001 absorbance unit/min.

### Phosphodiesterase activity

Phosphodiesterase activity (PDE) was determined by a modified method [23]. Fifty microliters of venom solution was added to 650 µL of an assay mixture containing 2.5 mM Ca-bis-p-nitrophenylphosphate, 0.01 M MgSO_4_ and 0.17 M veronal buffer (pH 9.0). Substrate hydrolysis was measured at 440 nm. One unit of PDE activity was defined as the amount of enzyme that caused an increase of 0.001 absorbance unit/min.

### Phosphomonoesterase activity

Phosphomonoesterase activity (PME) was determined by a modified method [23]. Fifty microliters of venom solution was added to 400 µL of an assay mixture containing 0.01 M p-nitrophenylphosphate in glycine buffer (pH 8.5). The mixture was incubated at 37°C for 30 min. At the end of the incubation period, 1 mL of 0.2 M sodium hydroxide was added and allowed to stand for 20 min. The absorbance of the mixture was measured at 440 nm. One unit of PME activity was defined as the amount of enzyme that caused an increase of 0.001 absorbance unit/min.

### Fibrinolytic activity

Fibrinolytic activity was determined by a modified method [24]. The fibrin clot plate was prepared by mixing 12 mL of 2% agarose with an equal volume of 1% bovine fibrinogen solution in 100 mm Petri dishes containing 0.08 mL of thrombin solution (10 Units/mL). The plate was placed for clotting at room temperature for 1 h. The holes (2 mm in diameter) were punched on the fibrin plate for venom application. Five microliters of the various concentration of venom solution were carefully dropped into each hole and incubated at 37°C for 17h. One unit of fibrinolytic activity was defined as the amount of venom (μg) producing a clear zone 10 mm in diameter on the fibrin plate. The activity is reported as μg venom/unit. 

*Fibrinogenolytic activity*

Fibrinogenolytic activity was determined by a modified method [24]. One hundred microliters of fibrinogen solution (2 mg/mL) with 20 µL of 0.45 mg/mL venom solution in 20 mM Tris-HCl buffer (pH 7.4) containing 0.1 M NaCl was incubated at 37°C. At various time points (0, 0.5, 1, 2, 4, 6 and 24 h), 10 μL aliquots of the reaction solution were mixed with an equal volume of sample buffer containing 4% β-mercaptoethanol, 4% SDS, 10% glycerol, 0.05% bromophenol blue and 50 mM Tris-HCl pH 6.8 then boiled at 100°C for 5 min and analyzed on 12.5% SDS-PAGE.

### Hyaluronidase activity

Hyaluronidase activity (HYA) was determined by a modified method [25]. One milliliter of the substrate containing 200 µg hyaluronic acid, 0.2 M acetate buffer (pH 5.0) and 0.15 M NaCl mixed with 100 µL of venom solution was incubated at 37°C for 30 min. The reaction was terminated by the addition of 2.5% cetyltrimethylammonium bromide in a 2% sodium hydroxide solution. The HYA activity was expressed as National Formula Unit per milligram (NFU/mg) at 400 nm absorbance.

### Arginine ester hydrolase activity

Arginine ester hydrolase activity (AEH) was determined by the spectrophotometric procedure [26]. Fifty microliters of venom solution (1 mg/mL) was mixed with 0.5 mL of p-toluene-sulfonylarginine methyl ester (0.4 mg/mL) as substrate. The reaction was read at 15-second intervals for 3 min at the absorbance of 247 nm. One unit of the AEH activity was defined as the amount of enzyme hydrolyzing 0.5 µmoles of substrate/min.

### Coagulant activity

Coagulant activity was determined by a modified method [27]. Twenty microliters of various venom concentrations was added to 200 µL of 2 mg/mL bovine fibrinogen dissolved in 50 mM Tris-HCl pH 7.5, incubated in a water bath at 37°C, and the clotting times were recorded. The minimum coagulant dose (MCD) is defined as the amount of venom (μg) inducing coagulation of plasma in 60 seconds. The activity is reported as μg venom per unit. 

### Determination of procoagulant activity and neutralization by antivenoms

The procoagulant activity of PKV was determined using human citrated plasma as substrate. Fresh human blood was collected into sodium citrate tubes and centrifuged at 1200 rpm for 10 min, then the plasma was aliquoted for further modified assay [28]. One hundred microliters of PKV sample (diluted in phosphate buffer saline to various concentrations) was added into 96-well microplates. Then, 100 µL of citrated human plasma (40 µL 0.4 M calcium chloride in 1 mL of citrated human plasma) was added simultaneously to the venom-containing wells. Coagulation activity was measured using the turbidimetric method [29]. The clots were formulated on real-time monitoring at 405 nm absorbance at 37°C for 15 min, using the microplate reader M965+ (Metertech., Taiwan). The time for plasma clotting was defined as the time when the absorbance reading was 0.02 U greater than the mean of the first two absorbance measurement. The minimum coagulation dose (MCD) was defined as the venom dose that induces substrate coagulation in 3 min. 

The neutralization was determined using PKV at the dose of 2 MCD each pre-incubated with various dilutions of antivenoms (HPAV, DSAV, TAAV and CRAV) at 37^o^C for 30 min. The total volume of the venom-antivenom mixture (1:1) was standardized at 100 µL. One hundred microliters of citrated human plasma was then added simultaneously into the 96-well microplate containing the venom-antivenom mixtures. The clotting time in the neutralization assay was then performed as described above. The effective dose (ED) was defined as the dose of antivenom (µL) that prolonged the clotting time of the citrated human plasma to three times that of the control (2 MCD of venom, without antivenom). The neutralizing capacity for venom procoagulant effect was expressed as an effective ratio in terms of venom amount (mg) neutralized by antivenom (mL) at the point corresponding to ED.

### Determination of hemorrhagic activity and neutralization by antivenom

The hemorrhagic activity was determined by intradermal injection of varying dilutions of PKV (30 µL) into the back skin of ICR mice (20-25g). Three mice were used per dose. Ninety minutes after injection, the mice were killed by isoflurane inhalation. Their skins were removed for evaluation of the diameters of the hemorrhagic areas. The minimum hemorrhagic dose (MHD) was defined as the amount of venom (µg) that causes a hemorrhagic spot of 10 mm in diameter. The median effective dose (ED_50_) is defined as the volume of antivenom (µL) that reduces the diameter of the hemorrhagic spot in animals by 50% when compared with the control group injected with venom/saline mixture. The challenge dose used was 2MHDs. Mixtures of a fixed amount of venom and various dilutions of antivenoms (HPAV, DSAV, TAAV and CRAV) were prepared and incubated at 37^o^C for 30 min, and aliquots of 30 µL were injected intradermally into the back skin of mice. The diameter of hemorrhagic lesions was quantified as described above. The neutralizing ability of antivenom was expressed as ED_50_ [30].

### Determination of molecular mass and protein components

Protein components were separated with relative molecular mass using sodium dodecyl sulfate polyacrylamide gel electrophoresis (SDS-PAGE) [31]. Venom (30 µg) was run under the non-reducing condition on 12.5% SDS-PAGE gel. A constant current of 30 mA was applied to the gel under the running buffer (25 mM Tris-glycine, 192 mM glycine and 0.1%SDS, pH 8.8). Thermo Scientific PageRuler Prestained Protein Ladder was used as molecular weight markers ranging from 10 to 130 kDa. The gel was stained with 0.2% Coomassie Brilliant Blue R-250 for 60 min and destained with the solvent (methanol: acetic acid: distilled water at the ratio of 25: 12.5: 62.5). 

### Western blotting

Following gel electrophoresis (SDS-PAGE) as described above, *P. kelomohy* venom was transferred to a PVDF membrane (Hybond-P, Amersham, USA) using ECL-Semi-Dry blotters (Amersham Biosciences UK) containing 25 mM Tris, 192 mM glycine and 20% v/v methanol, pH 8.3, using 40 mA for 2 h. The membrane was then soaked in 5% BSA in TBST (50 mM Tris-HCl pH 7.4 containing 0.15 M NaCl and 0.05% Tween-20) at 37^o^C for 1 h, and washed three times with TBST. The membrane was incubated with 1:100 dilutions of each antivenom (TAAV, DSAV, CRAV, and HPAV) in 3% BSA/TBST, at 4^o^C for 16 to 18 h. After washing with TBST, the membrane was incubated in 1:1000 dilution of rabbit anti-horse IgG conjugated with HRP (Sigma, USA) in 3% BSA/TBST at room temperature for 1 h, and then washed with TBST. The membrane was soaked in 0.6% 4-chloro-1-naphthol and 0.006% hydrogen peroxide in the dark container until the bands were developed. The reaction was stopped with water. The membrane was dried and recorded in the photo [32]. 

### Proteomic analysis

Crude PKV was mixed with lysis buffer containing 1% Triton X-100 (Merck, Germany), 1% sodium dodecyl sulfate (SDS) (Merck, Darmstadt, Germany), and 1% NaCl (Merck, Germany). Protein concentration was measured by the Quick Start™ Bradford Protein Assay (Bio-Rad, Berkeley, CA, USA). A total of 30 µg protein was separated by 12% SDS-PAGE (Bio-Rad, CA, USA). The gel bands were visualized by Coomassie R-250 solution (Bio-Rad, CA, USA). The gel was cut along the lane and subjected to tryptic digestion. Briefly, venom proteins were reduced by 4 mM dithiothreitol and alkylated by 250 mM iodoacetamine (Sigma-Aldrich, Saint Louis, MO, USA). All of the solution was removed and 0.1% trypsin solution (Sigma-Aldrich, USA, T6567) was added to the gel to perform tryptic digestion overnight. The supernatant peptide was dried using a centrifugal concentrator (TOMY, Katsushika, Japan). Then venom peptides were dissolved in 0.1% formic acid (Sigma-Aldrich, USA). The samples were evaluated by an Ultimate® 3000 Nano-LC system analyzer (Thermo Scientific, USA) coupled with a microTOF-Q II (Bruker, Bremen, Germany). The protein identification was performed using the software Mascot Daemon (Matrix Science, Boston, MA, USA) against the Chordata NCBI database, at a 95% significance threshold. The search parameters were set including one missed cleavage site, variable modifications of carbamidomethyl (C) and oxidation (M), 0.8 Da for MS peptide tolerance, and 0.8 Da for MS/MS tolerance due to the default parameter setting of the Mascot Daemon 2.2.2 software. Three biological replications were performed [33, 34].

### Statistical analysis

Each dilution of enzymatic activity was done in triplicate. The mean values are presented as mean ± SD. The results were evaluated by Analysis of Variances (ANOVA); the significant differences among groups were compared by Duncan’s multiple range test, with p < 0.05 indicating significance.

## Results

Two adult specimens of *P. kelomohy*, a male and a female, were collected from Chiang Mai Province. The size of snakes was recorded as follows: snout-vent length of 93.0 and 111.5 cm, total length of 110.5 and 131.0 cm, and bodyweight of 210 and 365 g for males and females, respectively. Venom was collected twice at a one-month interval from both snakes. The average fresh and dry venom weights were 131.48 and 31.60 mg/snake, respectively.

### Lethal toxicity and neutralization capacity by antivenom

The intravenous LD_50_ of PKV was 0.67 (0.58 - 0.78) µg/g mouse body weight. The efficacy values of each antivenom protecting 50% (ED_50_) of mice injected with 2-3 LD_50_ of PKV are summarized in Table 1. The neutralization capacity (ER_50_) was expressed as the amount of PKV (mg) neutralized by 1 mL of each antivenom at a 50% survival rate.

**Table 1.** The efficacy values (ED_50_) and neutralization capacities (ER_50_) of four antivenoms against *Protobothrops kelomohy* venom in mice

Antivenom	ED_50_ (µL of each antivenom against 3 LD_50_ of PKV)	Neutralization capacity (mg of PKV neutralized by 1 mL of each antivenom)
**HPAV**	39.70 (32.13-49.08)	1.02 (0.83-1.26)
**DSAV**	111.33 (85.23-145.41)	0.36 (0.28-0.48)
**TAAV**	293.38 (234.96 - 356.59)^*^	0.12 (0.09-0.14)
**CRAV**	^**^	^**^

^*^Antivenom against 2.5 LD_50_ of PKV

^**^Maximum of antivenom (CRAV) used (473 µL) against 2 LD_50_ of PKV cannot protect all mice.

### Enzymatic activities

Among the four venoms (PKV, TAV, CRV and DSV), all enzymatic activities exhibited marked quantitative differences (p < 0.05), except the comparable HYA activities between PKV (1.27 ± 0.44 NFU/mg) and CRV (1.97 ± 1.11 NFU/mg), which showed no significant difference (Table 2). PKV showed markedly higher (p < 0.05) PRO (0.115 ± 0.010 unit/mg/min), i.e., nearly 58 times, LAAO (3.77 ± 0.09 unit/mg/min) 2.7 times, PDE (1.25 ± 0.02 unit/mg/min) 1.6 times, PME (0.022 ± 0.001 unit/mg/min) 3 times, and AEH (1.22 ± 0.03 unit/mg/min) 8 times but substantially lower in PLA_2_ (96.25 ± 1.14 unit/mg/min) 2.4 times and HYA (1.27 ± 0.44 NFU/mg) 66 times than that of DSV. When compared with CRV, all enzymatic activities of PKV were significantly lower (p < 0.05); however, the PLA_2_ and PDE (1.25 ± 0.02 unit/mg/min) activities were about two-fold higher (p < 0.05). Meanwhile, the PRO, PLA_2_, LAAO, fibrinolytic (10.41 ± 0.82 µg**/**unit), and coagulant (MCD = 4.62 ± 0.17 µg**/**unit) activities of PKV were significantly higher by 1 to 4 times, but PDE, PME, HYA and AEH activities were 1 to 2 times significantly lower than that of TAV. The undetectable (UD) of fibrinolytic and coagulant activities of DSV was defined as the highest amount of DSV that failed to produce a clear zone of 10 mm in diameter on the fibrin plate and plasma coagulation in 60 seconds, respectively. 

**Table 2.** Enzymatic activities of *Protobothrops kelomohy* venom in comparison with the venoms of *Trimeresurus alobolabris*, *Calloselasma rhodostoma* and *Daboia siamensis*

Enzymatic activities	PKV	TAV	CRV	DSV
**PRO (unit/mg/min)**	0.115 ± 0.010^a^	0.074 ± 0.003^b^	0.222 ± 0.006^c^	0.002 ± 0.000^d^
**PLA_2_ (unit/mg/min)**	96.25 ± 1.14^a^	36.25 ± 0.79^b^	46.01 ± 0.53^c^	227.61 ± 1.71^d^
**LAAO (unit/mg/min)**	3.77 ± 0.09^a^	3.28 ± 0.08^b^	4.49 ± 0.06^c^	1.41 ± 0.03^d^
**PDE (unit/mg/min)**	1.25 ± 0.02^a^	1.40 ± 0.02^b^	0.63 ± 0.01^c^	0.79 ± 0.03^d^
**PME (unit/mg/min)**	0.022 ± 0.001^a^	0.051 ± 0.003^b^	0.107 ± 0.005^c^	0.007 ± 0.001^d^
**Fibrinolytic (µg/unit)**	10.41 ± 0.82^a^	44.02 ± 2.44^b^	1.81 ± 0.24^c^	UD
**HYA (NFU/mg)**	1.27 ± 0.44^a^	45.47 ± 2.75^b^	1.97 ± 1.11^a^	84.23 ± 3.70^c^
**AEH (unit/mg/min)**	1.22 ± 0.03^a^	2.67 ± 0.05^b^	2.49 ± 0.07^c^	0.15 ± 0.01^d^
**MCD (µg/unit)**	4.62 ± 0.17^a^	8.98 ± 0.63^b^	1.30 ± 0.05^c^	UD

^a,b,c,d^ Means within a row with different superscripts between groups of each measurement differ significantly (p < 0.05). PRO: protease activity; PLA_2_: phospholipase A_2_ activity; LAAO: L-amino acid oxidase activity; PDE: phosphodiesterase activity; PME: phosphomonoesterase activity; MCD: minimum coagulant dose; AEH: arginine ester hydrolase activity; HYA: hyaluronidase activity; UD: undetectable; PKV: *Protobothrops kelomohy* venom; DSV: *Daboia siamensis* venom*;* TAV: *Trimeresurus albolabris* venom; CRV: *Calloselasma rhodostoma* venom.

**Figure 1.**
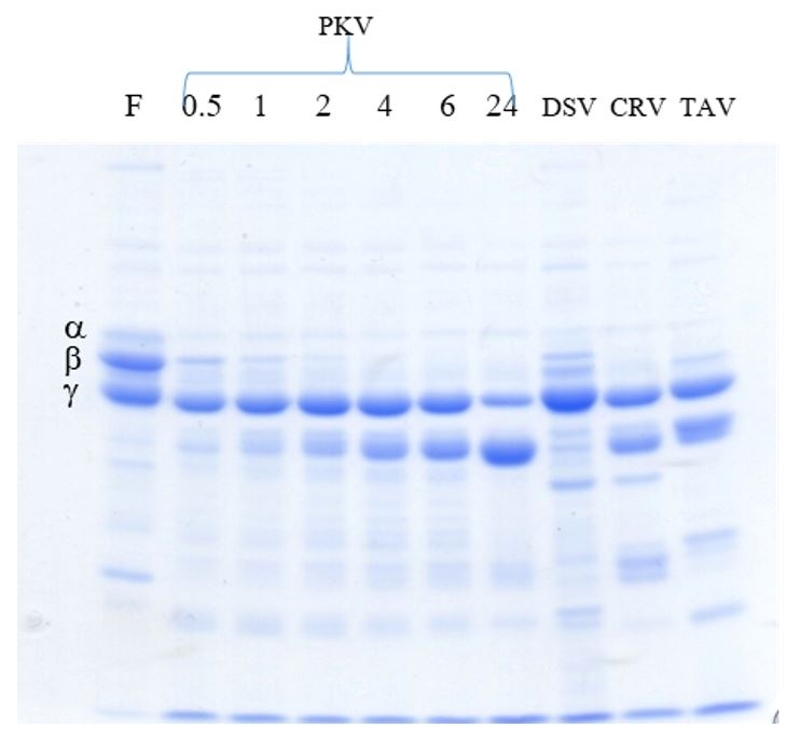
Fibrinogenolytic activity of *Protobothrops kelomohy* venom (PKV). PKV was incubated with fibrinogen (F) at various times (0, 0.5, 1, 2, 4, 6 and 24 h) in comparison with the other venoms incubated with fibrinogen at 24 h only. The bands corresponding to α, β and ɣ chains of fibrinogen (F) are labelled. DSV: *Daboia siamensis* venom*;* CRV: *Calloselasma rhodostoma* venom*;* TAV: *Trimeresurus albolabris* venom.

PKV showed the fibrinogenolytic activity on the α-chain and β-chain of fibrinogen (Figure 1). Cleavage of the α-chain and β-chain of fibrinogen by PKV began within 0.5 h, and was fully digested after 1 h. At 24 h, complete digestion of α-chain and β-chain was observed in PKV, CRV and TAV whereas DSV digested only α-chain of fibrinogen. None of the four venoms cleaved the ɣ-chain of fibrinogen. The experiment was performed in duplicate with similar results. 

### Procoagulant activity of PKV and neutralization by antivenoms

PKV exhibited procoagulant activity on human citrated plasma with a minimal coagulation dose (MCD) of 12.5 ± 0.016 µg/mL. The procoagulant effect of PKV was effectively neutralized by HPAV (2.89 ± 0.05 mg/mL) and TAAV (0.96 ± 0.08 mg/mL), whereas DSAV and CRAV were less effective (Table 3).

**Table 3.** Procoagulant activity of *Protobothrops kelomohy* venom and neutralization by HPAV, DSAV, TAAV and CRAV.

PKV		ED (mg/mL)			
MCD (µg/mL)	Challenge dose (2 MCD; µg/mL)	HPAV	TAAV	DSAV	CRAV
**12.50 ± 0.02**	25.00	2.89 ± 0.05^a^	0.96 ± 0.08^b^	< 0.33	< 0.33

^a,b^ The mean values for HPAV and TAAV using the Student’s *t* test showed significant differences (p < 0.001). MCD: minimum coagulation dose was defined as the amount of venom required to cause clotting of citrated human plasma in 3 min; ED: effective dose was defined as the dose of antivenom capable of prolonging the clotting time of challenge dose to 3 times that of the control. ED was expressed as the venom amount per unit volume of antivenom (mg/mL).

### Hemorrhagic activity of PKV and neutralization by antivenoms

PKV showed a minimal hemorrhagic dose (MHD) of 1.20 ± 0.71 µg/mouse. The neutralization was based on the median effective doses (ED_50_). The median effective dose (ED_50_) of HPAV against the hemorrhagic effect of PKV at 2 MHD (2.40 µg/mouse), namely 38.10 ± 2.69 mg/mL, was more effective than those of TAAV by 4 fold, DSAV by 41 fold and CRAV by 127 fold. Meanwhile DSAV and CRAV did not differ significantly in neutralizing the hemorrhagic effect of PKV (Table 4). 

**Table 4.** Hemorrhagic activity of *Protobothrops kelomohy* venom (PKV) and neutralization by HPAV, TAAV, DSAV and CRAV

PKV		ED_50_ (mg/mL)			
MHD (µg/mouse)	Challenge dose (2 MHD; µg/mouse)	HPAV	TAAV	DSAV	CRAV
**1.20 ± 0.71**	2.40	38.10 ± 2.69^a^	8.76 ± 1.25^b^	0.92 ± 0.06^c^	0.30 ± 0.02^c^

^a,b,c^ Means within a row with different superscripts between groups of each measurement differ significantly (p < 0.05). MHD: minimal hemorrhagic dose was defined as the amount of venom (µg) required to induce a hemorrhagic skin lesion of 10 mm diameter; ED_50_: median effective dose was defined as the dose of antivenom capable of reducing a venom’s hemorrhagic activity of 2MHD by 50%. ED_50_ was expressed as the venom amount per unit of antivenom volume (mg/mL).

### Molecular mass and protein components

**Figure 2.**
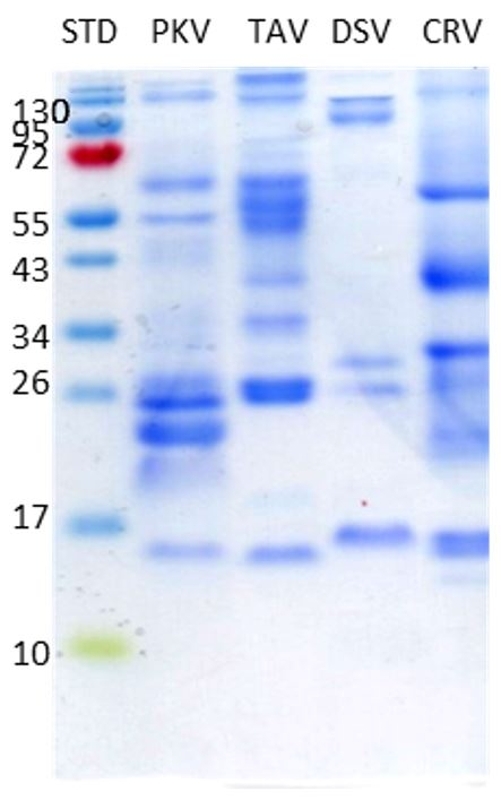
SDS-PAGE patterns of four snake venoms (30 µg/well each) under non-reducing conditions. STD: standard protein markers (Thermo scientific PageRuler Prestained Protein Ladder); PKV: *Protobothrops kelomohy* venom*;* TAV: *Trimeresurus albolabris* venom; DSV: *Daboia siamensis* venom*;* CRV: *Calloselasma rhodostoma* venom.

SDS-PAGE pattern under the non-reducing conditions of all four venoms revealed significant compositional differences (Figure 2). The overall marked dense protein bands differed in the region of molecular mass by 15 to more than 130 kDa. The protein pattern of PKV was distinct from the other viper venoms, which were TAV, DSV and CRV. 

### Specific proteins by western blot analysis

**Figure 3 .**
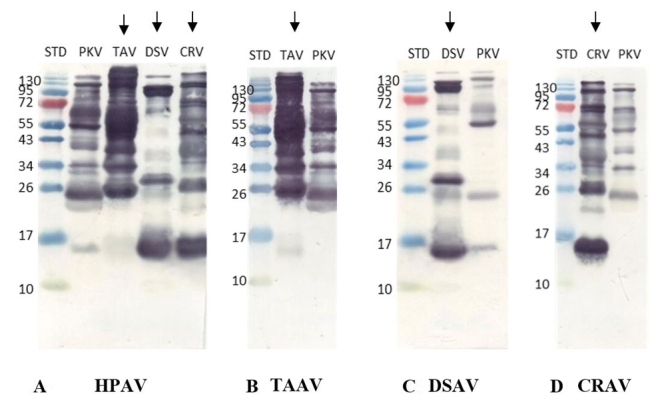
Western blot profiles of *Protobothrops kelomohy* venom exhibited cross-reactivity with various antivenoms. **(A)** Immunoblotting profile of four venoms with hemato polyvalent antivenom (HPAV). (**B)** Immunoblotting profile of TAV and PKV with *Trimeresurus albolabris* monovalent antivenom (TAAV). (**C)** Immunoblotting profile of DSV and PKV with *Daboia siamensis* monovalent antivenom (DSAV). **(D)** Immunoblotting profile of CRV and PKV with *Calloselasma rhodostoma* monovalent antivenom (CRAV). Arrow symbols represent the venom of snake species used for generating the antivenom. STD: standard protein; DSV: *Daboia siamensis* venom; CRV: *Calloselasma rhodostoma* venom; TAV: *Trimeresurus albolabris* venom; PKV: *Protobothrops kelomohy* venom.

The venom-antivenom interaction was investigated by western blotting analysis to identify the venom proteins that specifically bind to each antivenom. PKV, along with TAV, DSV and CRV, was used to check the cross-reactivity against homologous and heterologous antivenoms (HPAV, TAAV, DSAV and CRAV). Immunoblotting profiles of all four venoms with HPAV revealed binding of the antibodies to venom proteins of DSV and CRV at a molecular mass of approximately 15 to 130 kDa, and binding to PKV and TAV venom proteins at 26 to 130 kDa and above (Figure 3A). The immunoreactivity of PKV and TAAV was predominantly at the venom protein bands of 20 - 130 kDa (Figure 3B). The DSAV and CRAV showed weak binding to all venom protein bands of PKV (Figure 3C and 3D). 

### Proteomic analysis

**Figure 4.**
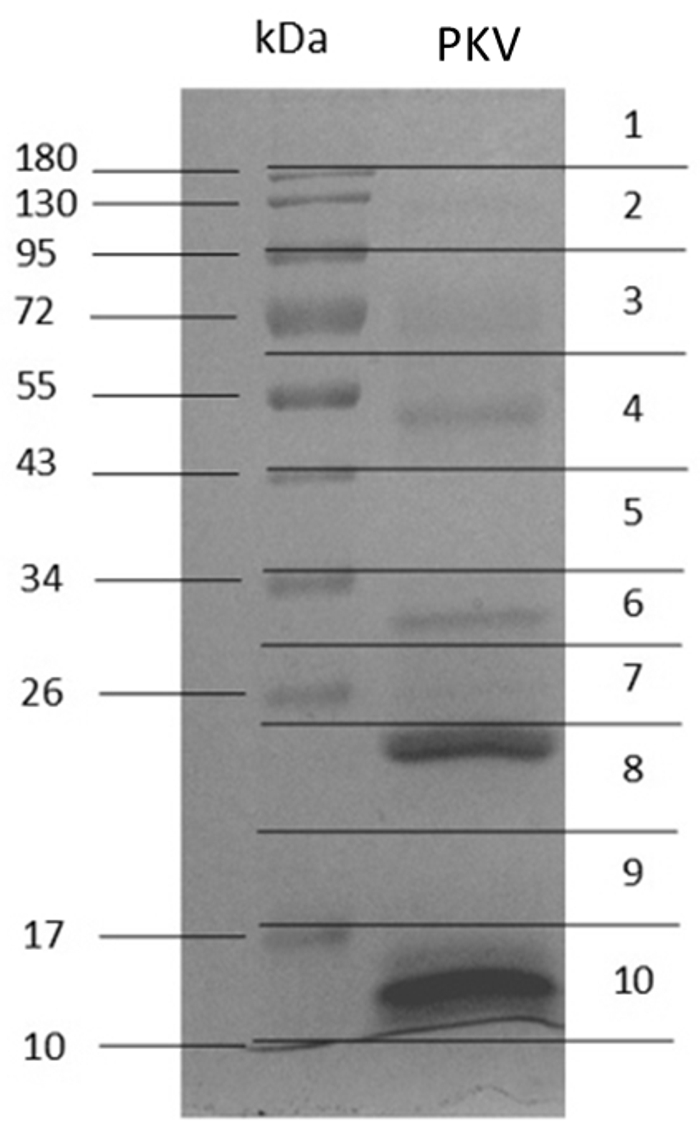
Protein separation of *Protobothrops kelomohy* venom on Coomassie blue-stained 12% gel electrophoresis under reducing conditions.

The Coomassie blue-stained 12% gel electrophoresis of PKV under reducing conditions revealed two intense protein bands with molecular mass ranging from 26 to 180 kDa including multiple faint bands (Figure 4). Molecular masses ranging from 10 to 25 kDa demonstrated two strong bands (Figure 4). The entire PKV gel lane was divided into 10 pieces (1-10) to perform tryptic digestion and protein identification. 

**Figure 5.**
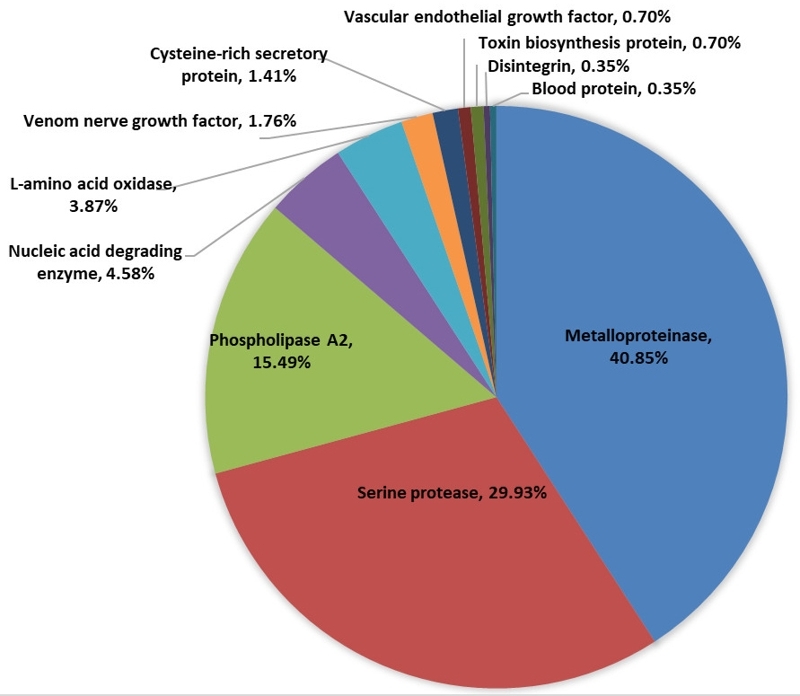
The classification of *Protobothrops kelomohy* venom proteins.

A total of 412 proteins were identified from PKV (Additional file 1, Additional file 2). The protein classification was performed according to functions (Figure 5). Eleven protein classes were found in PKV including metalloproteinase (40.85%), serine protease (29.93%), phospholipase A_2_ (15.49%), 5 ~ -nucleotidase (4.23%), L-amino acid oxidase (3.87%), venom nerve growth factor (1.76%), cysteine-rich secretory protein (1.41%), vascular endothelial growth factor (0.70%), toxin biosynthesis protein (0.70%), disintegrin (0.35%), phosphodiesterase (0.35%) and blood protein (0.35%). Metalloproteinase (40.85%), serine protease (29.93%) and phospholipase A_2_ (15.49%) were the major protein classes in PKV.^2^

**Figure 6.**
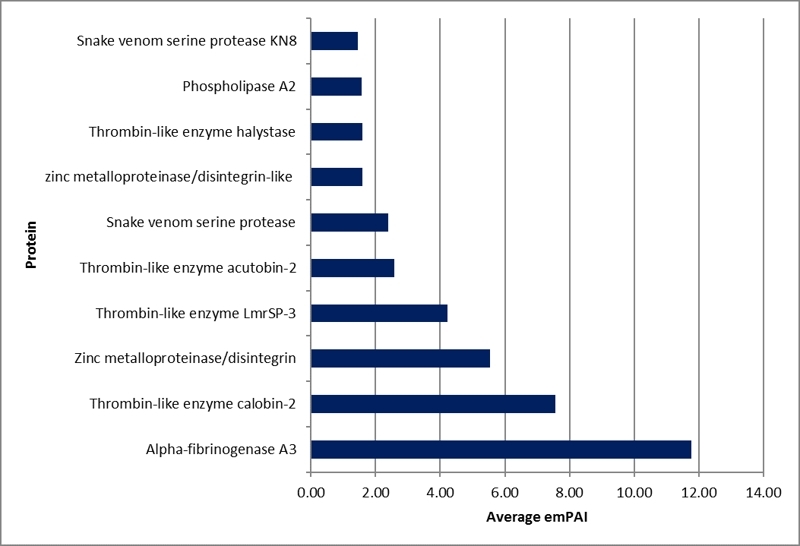
The top-ten most abundant proteins in *Protobothrops kelomohy* venom (PKV).

Proteomics not only provided the protein identification information but also offered quantification through the Exponentially Modified Protein Abundance Index (emPAI). The top-ten most abundant proteins were ranked by emPAI value (Figure 6). Alpha-fibrinogenase A3, thrombin-like enzyme calobin-2 and zinc metalloproteinase/disintegrin were observed in remarkably large quantities in PKV. In addition, several thrombin-like enzymes were highly prominent in the venom.

## Discussion

Snake venoms may present interspecific variation in protein compositions due to species differences. The knowledge of species variation among pit viper venoms is essential to understand the potential variable toxicity and dissimilar antigenicity of the venom proteins. The present work unveiled the venom proteomics and biochemical properties of PKV including the cross-neutralization of four antivenoms available in Thailand against the lethal toxicity, procoagulant and hemorrhagic effects of PKV. It has been reported that polyvalent antivenom should be considered in the victims bitten by little known or new venomous snake species [35]. However, the limitations on cross-reactivities associated with interspecific venoms between polyvalent and monovalent antivenom therapy are unclear [36, 37]. The neutralizing capacity of any antivenom is restricted to cross-reaction with similar toxins in specific venom used for immunization [38, 39]. The present results revealed that HPAV provided the most potent efficacy in neutralizing against the lethal toxicity of PKV at 1.02 mg/mL in mice. However, all of the monovalent antivenoms raised specifically against the venoms of *D. siamensis* (DSAV)*, T. albolabris* (TAAV) and *C. rhodostoma* (CRAV) showed either ineffectiveness or low effectiveness in neutralizing capacity against PKV (Table 1). The cross-neutralization of these antivenoms might limit their reaction with the toxin compounds in PKV that are not included in the immunized venom mixture. The antigenicity of abundant venom proteins in DSV, TAV and CRV might be the synergized formula in producing HPAV, by which the higher quantities of PLA_2_ and HYA activities in DSV including the PDE, PME, AEH and HYA activities in TAV, and the PRO, PME, AEH, fibrinolytic and coagulant activities in CRV are expected to improve the neutralizing potency of HPAV higher than those of DSAV, TAAV and CRAV. Meanwhile, the known habitat of *P. kelomohy* is only in Thailand and hence, the priority choice of antivenom treatment for its envenomation should be that available in the country. Many reports investigated the cross-neutralization of TAAV with various snake venoms of *Trimeresurus sp.* and *Protobothrops sp.* [8, 9, 19, 28, 35, 40]. An *in vivo* study using TAAV against the heterologous venoms of four Indonesian *Trimeresurus* species confirmed that the closer phylogenetic relationship of interspecific snakes provided better cross-neutralizing capacity [28]. It is interesting for further study on the cross-neutralizing capacity of PKV by *Protobothrops* (*Trimeresurus*) *mucrosquamatus* and *Trimeresurus stejnegeri* bivalent antivenom produced in Taiwan, which has been revealed to be effective not only against the lethal toxicity but also against the hemorrhagic, necrotizing and thrombin-like activities of heterologous *Trimeresurus* venoms [35], some of which presently are revised to the genus *Protobothrops*.

Since the PKV genome is not available and this study used the Chordata database for protein identification, the total number of 412 proteins could include redundant hits from the similar proteins of different organisms. The exact number of proteins presented in PKV might be lower. According to the proteomics analysis, PKV consists of analogous composition, but the difference in quantity, to the venoms of *P. mucrosquamatus* (PMV) and *P. flavoviridis* (PFV). The main protein classes of PKV were SVMP (40.85%), SVSP (29.93%) and PLA_2_ (15.49%) (Figure 5), whereas those of PMV were SVMP (> 40%) and PLA_2_ (about 25%), while PFV comprised the largest portions of PLA_2_ (55.14%) and SVMP/DI (31.34%). Meanwhile, the SVSP is more abundant (> 10%) in PMV than in PFV [41]. Mass spectrometry identified proteins that corresponded to the observed enzyme activities. Alpha-fibrinogenase A3 (Q9PRW2.1) was the most abundant protein in PKV (Figure 6). The fibrinolytic activity of PKV was 10.41 ± 0.82 µg**/**unit, which was higher than those of CRV and DSV (Table 2). In *P. mangshanensis*, delayed coagulopathy was found in the victim. Fibrinogen decreased to 121 mg/dL and D-dimer increased to 377 ng/mL over 24 h [9]. This delayed coagulopathy might have resulted from the high amount and activity of *Protobothrops* fibrinogenase. Another enzyme copiously presented in PKV was proteinase. Protease activity at 0.115 ± 0.010 unit/mg/min of PKV was exhibited by several proteinases in the venom. Four proteases (O93517.1, P0DMH6.1, XP_015685695.1 and Q71QH5.1) were highly expressed among the top-ten most common proteins in PKV. This finding was similar to *P. flavoviridis*, which showed protease as a major component in venom [42]. PLA_2_ (P0CV89.1) ranked ninth among the most abundant proteins in PKV. Several forms of PLA_2_ were found in the PKV including O42187.2, P14418.1, P70088.1 and CAA54363.1. This result agreed with *P. flavoviridis*, half of whose venom was composed of different PLA_2_s [41]. The venom protein profile was strongly correlated with the enzyme activities of PKV. 

The present study indicates that the venoms from different snake species were variable in composition (Figure 2), a variability that may account for the discrepancy in toxicity with cross-neutralization of different antivenoms’ efficacy [38, 39]. The PKV likely share cross-neutralization with a similar protein and antigenicity profile, despite the disjunctive species among the vipers (Figure 3). Hence, the current study aimed to investigate the protein composition of the PKV through a decomplexing proteomic approach and compare it with the other viperid venoms by examining the immunorecognition of the various protein components in the venoms by antivenoms. The HPAV exhibited a similar binding profile toward the protein bands of PKV and the other three viperid venoms (Figure 3A). The immunoreactivity of TAAV was distinctive as to the venom protein bands of PKV ranging from 20 to 130 kDa (Figure 3B). However, the PKV venom protein bands at 15 to 130 kDa presented weak binding to DSAV (Figure 3C). The CRAV, on the other hand, showed poor immunorecognition of venom protein bands of PKV detected below 130 kDa (Figure 3D). 

The present findings reveal that most protein families (i.e. protease, PLA_2_, LAAO and PDE) were well conserved among viperid snakes despite their wide disjunctive distribution [39, 43, 44]. Nevertheless, the protein profiles among viperid venoms were not identical, due to variations observed in the presence of proteases, PLA_2_, LAAO, PDE and HYA and fibrinolytic activities and the expression level of proteomic analysis. The finding suggests the sharing of common protein epitopes among the different snake venoms. Consistently, the highest immunoreactivity was shown in HPAV, which had predominance of protease, LAAO, PLA_2_ and large proteins such as LAAO and PDE. Proteins with large molecular size are usually more antigenic and hence exhibit a better immunorecognition profile as the antivenom binds more effectively to the protein antigens available. The low-molecular-weight proteins such as disintegrin, a non-enzymatic protein in snake venom, may exhibit a lower immunological binding activity with antivenom presumably due to limited epitopes and antigenicity.

The immunoblotting analysis revealed that TAAV effectively recognizes other potent and abundant venom components of *P. kelomohy* (Figure 3B), including the enzymatic activities of PLA_2_, proteases, LAAO and PDE (Table 2). The similarity in venom protein family representation in *P. kelomohy* venom, and that of *T. albolabris* venom utilized in TAAV production, is reflected in the immunoreactivity of this antivenom. Comparing the levels of immune recognition gathered from both DSAV and CRAV antivenoms with the cross-neutralization capacity is not straightforward, due to a moderate immunocapturing capability of DSAV and CRAV to venom components from *P. kelomohy*. The results indicate the opposite relationship for *P. kelomohy*, with overall protease, LAAO and PDE activities being lower and PLA_2_ activity being higher in DSV (Table 2). It should be noted, however, that hematotoxic polyvalent antivenom activity against the venoms from viperid snakes appeared to be efficiently immunocaptured during the *in vivo* experiment, but western blot analysis indicated that it was recognized by hematotoxic polyvalent antivenom as well as by the specific antibody (Figure 3A). 

Hematotoxic snake envenoming causes consumption coagulopathy, and consequently can be life-threatening in severe cases [45]. Toxin compositions in snake venoms vary by genera, species, age, sex and geography [44, 46-49]. The venom proteome of PKV presented a majority of protease, SVMP and SVSP, which are related to coagulophic effects such as hemorrhagic, fibrinogenolytic and prothrombin-activating activities [50-52]. In the present study, PKV showed fibrinogenolytic activity on the α-chain and β-chain of fibrinogen (Figure 1) indicating the presence of alpha and beta fibrinogenase in the venoms [53]. Clinical symptoms of *P. kelomohy* envenomation comprise extensive swelling without hemorrhagic bleb, including the suspected compartment syndrome at the bite site. The systemic effects involve coagulopathy and rhabdomyolysis. The effect of HPAV treatment of this envenoming victim was inconclusive [54]. The clinical symptoms in this case report are correlated with the major venom toxins in PKV, including the high enzymatic activities of protease, phospholipase, phosphodiesterase, fibrinolytic and procogulant (on bovine fibrinogen) (Table 2). The present study on neutralzing capacity of HPAV has found significant effectiveness against lethal toxicity, including the procoagulant (Table 3) and hemorrhagic effects of PKV (Table 4). Antivenom is the most effective in snake bite therapy if delivered promptly [39]. Thus, the possibilities for ineffective HPAV might include misidentified snake species leading to an inappropriate first antivenom choice or delayed antivenom therapy upon arrival at the hospital. 

## Conclusion

Enzymatic activities of PKV are quantitatively similar to other viperid venoms in relation to hematotoxic properties. The differences in venom proteomic composition are reflected in the efficacy of paraspecific antivenoms used for the therapeutics of *Protobothrops kelomohy* envenoming. Three major toxins in snake venom comprised of metalloproteinase, serine protease and phospholipase A_2_ are usually responsible for mediating the coagulopathic effect in viperid envenoming including *P. kelomohy*. Although specific antivenom for *P. kelomohy* presently is not available, the hematotoxic polyvalent antivenom showed the most potency in cross-neutralizing the lethal toxicity including procoagulant and hemorrhagic effects of PKV. 

## Supplementary material

The following online material is available for this article:

Additional file 1. Mascot report of protein identification.

Additional file 2. Mascot generic files of mass spectrometry analysis. Thirty Mascot generic files (.mgf) were obtained from three replications. Each *Protobothrops* replication contained ten LC-MS/MS results.
