# Supplementary material for: Biochemical and proteomic analyses of venom from a new pit viper, Protobothrops kelomohy
Source: J Venom Anim Toxins Incl Trop Dis. 2022 Apr 11;28:e20210080. doi: 10.1590/1678-9199-JVATITD-2021-0080 (PMC9005077; doi:10.1590/1678-9199-JVATITD-2021-0080)
Supplement: Additional file 2. [file 1678-9199-jvatitd-28-e20210080-s2.zip › jvatitd-2021-0080_suppl2_20220324/jvatitd-2021-0080_suppl2.pdf]

J Venom Anim Toxins incl Trop Dis
https://doi.org/10.1590/1678-9199-JVATITD-2021-0080

Supplementary Material to "Biochemical and proteomic analyses of venom from a new pit viper, *Protobothrops kelomohy*"

Additional file 2. Mascot generic files of mass spectrometry analysis. Thirty Mascot generic files (.mgf) were obtained from three replications. Each Protobothrops replication contained ten LC-MS/MS results.
